# Diagnosis of Schizophrenia Based on the Data of Various Modalities: Biomarkers and Machine Learning Techniques (Review)

**DOI:** 10.17691/stm2022.14.5.06

**Published:** 2022-09-29

**Authors:** M.G. Sharaev, I.K. Malashenkova, A.V. Maslennikova, N.V. Zakharova, A.V. Bernstein, E.V. Burnaev, G.S. Mamedova, S.A. Krynskiy, D.P. Ogurtsov, E.A. Kondrateva, P.V. Druzhinina, M.O. Zubrikhina, A.Yu. Arkhipov, V.B. Strelets, V.L. Ushakov

**Affiliations:** Senior Researcher; Skolkovo Institute of Science and Technology (Skoltech), Territory of Skolkovo Innovation Center, Bldg 1, 30 Bolshoy Boulevard, Moscow, 121205, Russia; Department Senior Researcher; N.A. Alekseyev Psychiatric Clinical Hospital No.1, 2 Zagorodnoye Shosse, Moscow, 117152, Russia;; Head of the Laboratory of Molecular Immunology and Virology; National Research Center “Kurchatov Institute”, 1 Akademika Kurchatova Square, Moscow, 123182, Russia; Senior Researcher, Laboratory of Clinical Immunology; Federal Research and Clinical Center of Physical-Chemical Medicine, Federal Medical Biological Agency of Russia, 1A Malaya Pirogovskaya St., Moscow, 119435, Russia;; Researcher, Laboratory of Human Higher Nervous Activity; Institute of Higher Nervous Activity and Neurophysiology, Russian Academy of Sciences, 5A Butlerova St., Moscow, 117485, Russia;; Head of the Laboratory for Fundamental Research Methods, Research Clinical Center of Neuropsychiatry; N.A. Alekseyev Psychiatric Clinical Hospital No.1, 2 Zagorodnoye Shosse, Moscow, 117152, Russia;; Professor, Professor of the Center of Applied Artificial Intelligence; Skolkovo Institute of Science and Technology (Skoltech), Territory of Skolkovo Innovation Center, Bldg 1, 30 Bolshoy Boulevard, Moscow, 121205, Russia;; Associate Professor, Professor of the Center of Applied Artificial Intelligence; Skolkovo Institute of Science and Technology (Skoltech), Territory of Skolkovo Innovation Center, Bldg 1, 30 Bolshoy Boulevard, Moscow, 121205, Russia;; Junior Researcher, Laboratory for Fundamental Research Methods, Research Clinical Center of Neuropsychiatry; N.A. Alekseyev Psychiatric Clinical Hospital No.1, 2 Zagorodnoye Shosse, Moscow, 117152, Russia;; Researcher, Laboratory of Molecular Immunology and Virology; National Research Center “Kurchatov Institute”, 1 Akademika Kurchatova Square, Moscow, 123182, Russia;; Researcher, Laboratory of Molecular Immunology and Virology; National Research Center “Kurchatov Institute”, 1 Akademika Kurchatova Square, Moscow, 123182, Russia;; PhD Student; Skolkovo Institute of Science and Technology (Skoltech), Territory of Skolkovo Innovation Center, Bldg 1, 30 Bolshoy Boulevard, Moscow, 121205, Russia;; PhD Student; Skolkovo Institute of Science and Technology (Skoltech), Territory of Skolkovo Innovation Center, Bldg 1, 30 Bolshoy Boulevard, Moscow, 121205, Russia;; PhD Student; Skolkovo Institute of Science and Technology (Skoltech), Territory of Skolkovo Innovation Center, Bldg 1, 30 Bolshoy Boulevard, Moscow, 121205, Russia;; Researcher, Laboratory of Human Higher Nervous Activity; Institute of Higher Nervous Activity and Neurophysiology, Russian Academy of Sciences, 5A Butlerova St., Moscow, 117485, Russia;; Chief Researcher, Laboratory of Human Higher Nervous Activity; Institute of Higher Nervous Activity and Neurophysiology, Russian Academy of Sciences, 5A Butlerova St., Moscow, 117485, Russia;; Associate Professor, Chief Researcher, Institute for Advanced Brain Research; Lomonosov Moscow State University, 27/1 Lomonosov Avenue, Moscow, 119192, Russia; Head of the Department; N.A. Alekseyev Psychiatric Clinical Hospital No.1, 2 Zagorodnoye Shosse, Moscow, 117152, Russia; Senior Researcher; National Research Nuclear University MEPhI, 31 Kashirskoye Shosse, Moscow, 115409, Russia

**Keywords:** MRI/fMRI in schizophrenia, EEG in schizophrenia, immunology in schizophrenia, biomarkers of schizophrenia, interpretable machine learning models, machine learning in diagnosing

## Abstract

Schizophrenia is a socially significant mental disorder resulting frequently in severe forms of disability. Diagnosis, choice of treatment tactics, and rehabilitation in clinical psychiatry are mainly based on the assessment of behavioral patterns, socio-demographic data, and other investigations such as clinical observations and neuropsychological testing including examination of patients by the psychiatrist, self-reports, and questionnaires. In many respects, these data are subjective and therefore a large number of works have appeared in recent years devoted to the search for objective characteristics (indices, biomarkers) of the processes going on in the human body and reflected in the behavioral and psychoneurological patterns of patients. Such biomarkers are based on the results of instrumental and laboratory studies (neuroimaging, electro-physiological, biochemical, immunological, genetic, and others) and are successfully being used in neurosciences for understanding the mechanisms of the emergence and development of nervous system pathologies.

Presently, with the advent of new effective neuroimaging, laboratory, and other methods of investigation and also with the development of modern methods of data analysis, machine learning, and artificial intelligence, a great number of scientific and clinical studies is being conducted devoted to the search for the markers which have diagnostic and prognostic value and may be used in clinical practice to objectivize the processes of establishing and clarifying the diagnosis, choosing and optimizing treatment and rehabilitation tactics, predicting the course and outcome of the disease.

This review presents the analysis of the works which describe the correlates between the diagnosis of schizophrenia, established by health professionals, various manifestations of the psychiatric disorder (its subtype, variant of the course, severity degree, observed symptoms, etc.), and objectively measured characteristics/quantitative indicators (anatomical, functional, immunological, genetic, and others) obtained during instrumental and laboratory examinations of patients.

A considerable part of these works has been devoted to correlates/biomarkers of schizophrenia based on the data of structural and functional (at rest and under cognitive load) MRI, EEG, tractography, and immunological data. The found correlates/biomarkers reflect anatomic disorders in the specific brain regions, impairment of functional activity of brain regions and their interconnections, specific microstructure of the brain white matter and the levels of connectivity between the tracts of various structures, alterations of electrical activity in various parts of the brain in different EEG spectral ranges, as well as changes in the innate and adaptive links of immunity.

Current methods of data analysis and machine learning to search for schizophrenia biomarkers using the data of diverse modalities and their application during building and interpretation of predictive diagnostic models of schizophrenia have been considered in the present review.

## Introduction

The World Health Organization considers schizophrenia as a socially significant mental disorder leading, if not treated, to severe forms of disability. Schizophrenia affects about 1–4% of the population according to various data, and only 31.1% of them in the world receive specialized psychiatric care [1]. Difficulty of statistical assessments of morbidity rate in schizophrenia is due to inability to register subclinically running forms of this disease causing no disadaptation using official statistics. Differentiation of schizophrenia from the diseases with a similar clinical picture is also among the problems arising in the process of providing care to these patients.

Medicine of the XXI century is characterized by widening of the spectrum of instrumental and laboratory studies used at all stages of diagnosis, treatment, and rehabilitation of patients. For example, neuroimaging examinations entered firmly the clinical practice having revolutionized the approaches to the diagnosis of the diseases almost in all fields of medicine including neurology. Owing to MRI and CT, these approaches have acquired a unique experience of clinical and neuroimaging correlations.

In clinical practice, various biomarkers, identified by the results of instrumental and laboratory examinations, are used for a large number of nosologies in diagnosis and verification of the disease stage, choice of treatment and rehabilitation tactics, and making a long-term prognosis. During the transition to the era of personalized medicine, identification of neuroimaging-based and molecular biomarkers, especially related to molecule-genetic factors, is important.

The development of new mathematical methods of analyzing the results of instrumental and laboratory studies as well as the progress in the field of machine learning and artificial intelligence, have led to the rapid integration of computer programs and information systems into research projects and clinical practice. Machine learning algorithms represent mathematical models designed to study patterns in experimental data in order to make prognoses based on new information. The advantage of machine learning techniques is their ability to take into consideration spatial correlations in the data of one modality (which, for example, makes it possible to discover weak and spatially distributed effects in the brain according to MRI findings) and also to combine and analyze data of different modalities and latent dependencies in them. Frequently, the problem lies in the absence of data in one of the modalities in some part of a sample, making classical analysis of differences between the groups impossible. Therefore presently, methods which allow one to work with multimodal data having skips for separate modalities have been developed. Moreover, while the results of classical statistics explain the group differences, machine learning models allow one to draw statistical conclusions at the level of an individual, which can help in making individual diagnostic or predictive decisions.

However, implementation of these general medical trends into clinical psychiatric practice ran against some objective difficulties. The attempts to supplement subjective results of the specialized psycho-pathologic examination of patients by psychiatrists with objective indicators of the patient’s condition obtained by instrumental and laboratory investigations are complicated by the fact that there is uncertainty in the understanding of pathophysiology of various mental disorders in psychiatry itself. The majority of biomarkers found by the present time, showing significant correlations with behavioral and other psychoneurological manifestations, are of low clinical value determined in terms of sensitivity, specificity, and prognostic significance. Identified cause-and-effect relations between biological markers and symptoms have not been yet established at the required level of evidence, and for these reasons application of biomarkers is not included into the existing clinical recommendations and treatment protocols. Lack of reliable “unimodal” biomarkers in psychiatry determines the importance of search for “multimodal” biomarkers based on consolidation of a wide spectrum of neuroimaging, electrophysiological, biochemical, immunological, genetic, and other data, although a large volume, a high dimensionality (hundreds of thousands and millions of variables), and heterogeneity of multimodal laboratory data make it difficult to integrate all available modalities within the frames of one investigation. Therefore, interest is growing in the clinician community to the modern methods of heterogeneous data integration using machine learning and deep learning techniques.

## Materials and Methods

**The aim of the present work** is:

to overview clinical tasks facing schizophrenia management which can be solved by finding appropriate biomarkers allowing objectivization of diagnosis establishment and clarification processes, choice and optimization of treatment and rehabilitation tactics, prediction of the disease course and outcome;

to overview the works devoted to finding the correlates between the results of clinical observations and neuropsychological testing and findings of the instrumental and laboratory studies performed;

to overview the current methods of machine learning, analysis, and consolidation of data allowing one to select diagnostically and prognostically significant biomarkers from the multimodal results of instrumental and laboratory studies for their application in clinical practice of schizophrenia treatment.

### Structure of the article

*Section 1* includes the review of the burning clinical goals of differential diagnosis of schizophrenia, the solution of which requires clinically valuable biomarkers permitting objectivization of medical decision-making process. The section also presents a brief overview of the instrumental and laboratory studies being carried out, the results of which are employed for the search for clinically significant biomarkers of schizophrenia.

*Section 2* is the main part containing the overview of the methods and results obtained therewith in the way of searching for correlates between clinical manifestations of schizophrenia and the data of instrumental and laboratory studies. The results considered in this section are classified according to the types of the data used (structural and functional MRI, EEG, immunological data) and the data of the clinical picture of the mental disorder (etiology and pathogenesis of the mental disorder, the type and subtype of schizophrenia, observed symptoms), which may be subjectivized by means of the detected biomarkers.

*Section 3* is devoted to the methods of data analysis and machine learning used in the task of searching for schizophrenia biomarkers including novel effective methods that have appeared in recent years and which allow consolidation of biomedical data of various modalities. The summary contains a list of currently important directions of investigations in the field of machine learning and data analysis, the solution of which is promising for implementation of the research findings into clinical practice of diagnosis, treatment, and post-treatment rehabilitation.

## *Section 1*. The main tasks in schizophrenia diagnosis

The tasks in the diagnosis of schizophrenia may be divided into fundamental and practical (clinical, or routine).

The following is referred to the fundamental tasks:

establishing the causes and mechanisms of schizophrenia development or its syndromes and symptoms;

development of reliable methods of early diagnosis.

Clinical (routine) tasks include:

determining the risk for developing schizophrenia;

assessment of prognostic outcomes (favorable or unfavorable);

establishing personalized targets for prevention, therapy, and rehabilitation.

Presently, in addition to the analysis of the test and questionnaire results, these goals may be solved by instrumental and laboratory examinations (structural MRI of the brain in various modes showing its anatomy; functional MRI and EEG reflecting functional alterations), data derived from the investigations of parameters of innate and adaptive immunity, and other studies may also be used.

Solving the main problems of schizophrenia diagnosis is associated with some methodological aspects of studying this pathology. Let us consider some of them.

### Agreement between diagnostic criteria of schizophrenia

Over a hundred years ago, Emil Kraepelin differentiated dementia praecox, later renamed schizophrenia, from manic-depressive psychosis, called later bipolar disorder. This paradigm of nosologic (implying existence of two different diseases each having unique etiopathogenetic mechanisms, therapeutic approaches, and outcome prognoses) separation of schizophrenia from affective disorders is the basis of categorical approach to the classification of psychic disorders, realized in all variants of international classifications of the disease right up to the tenth revision ICD-10 (World Health Organization, 1993). Predominance of the Kraepelin’s paradigm favored the consensus in the process of differentiation of various psychic disorders. Based on the standard criteria for this disorder, the agreement of the diagnosis of schizophrenia made by two mental health professionals reaches 90% and remains unchanged in the majority of cases for several years of observation of the same patient [2].

However, the experience of fundamental investigations in the XX century has demonstrated that the results of the works carried out on the basis of ICD criteria do not accept unambiguous conclusions or interpretations and are not verified in the attempt to replicate them. It is explained by two clinical-diagnostic dilemmas. For example, search for biomarkers is being carried out for the patients with schizophrenia whose condition matches the criteria of section F2 according to ICD-10, although clarification of specific features of these conditions is not conducted. Meanwhile, this section includes a wide spectrum of discrete pathologies varying in clinical-dynamic characteristics^1^.

There exists another diagnostic dilemma: K. Schneider’s first-rank symptoms of schizophrenia — echo of thoughts, thought alienation, and intraprojective auditory delusional perception — are always inherent for paranoid schizophrenia (F20.3) but are not encountered in simple pseudoneurotic and psychopathy-like schizophrenia (F20.6, F21.3, and F21.4, respectively).

In other words, the state of the same patient may correspond to the criteria of several disorders or not to meet the criteria of a specific disorder from the point of view of categorical approach. Consequently, orientation solely to the categorical approach, i.e. ICD criteria, when forming samples of patients with schizophrenia, may lead to the creation of a sample with very heterogeneous disorders.

These dilemmas become a starting point for initiative proposals to abolish the term “schizophrenia” excluding it from the list of diseases [3-5].

### Some aspects of phenomenological similarity of schizophrenia symptoms

This problem can be analyzed using as an example similarity and difficulty in distinction between negative symptoms and cognitive disorders in schizophrenia, especially in the context of their decisive influence on the quality of everyday functioning, social and professional realization of patients. Thus, for example, reduction of performance capability due to growing inattention can be regarded as both negative symptoms and cognitive deficit, or may be considered as “through” or “continuous” manifestation of schizophrenia causing asociality [6]. On the other hand, asociality may be caused by the reduction of speech fluency (i.e. incapability of operatively retrieving information from memory) underlying paralogicality of thinking and decrease of social competence level [7]. Evidence of phenomenological intersections is also proved by the fact that almost all patients with schizophrenia have cognitive impairment but negative symptoms are not detected in all of them [8], and difficulties in performing diagnostic test tasks are caused by the will decline or aberrations of goal setting [9, 10]. Similar inferences are also appropriate when comparing negative symptoms and phenomena of conceptual disorganization [3] or cognitive impairment and signs of depression [11].

### Continuum of symptoms and conventionality of boundaries between disorders

The aforementioned phenomenological intersections (similarity of manifestations) of symptoms of schizophrenia are complicated by the fact that each of them may be displayed with various degree of severity or intensity: from extremely severe forms up to persistence at the subsyndromal level [12-14], that, in a certain sense, “deletes the boundaries” between the categories of psychiatric disorders in general and, for example, between schizophrenia and bipolar disorder, in particular.

Dimensional approach, as an alternative to the categorical one, seems to be more accurate for solving both fundamental and practical issues of psychiatry. Based on factor analysis of the main manifestations of schizophrenia spectrum disorders, dimensions (domains) of their phenomenology or symptoms have been distinguished, which include positive disorders or distortion of reality perception (delusion and hallucinations), negative disorders or psychomotor impoverishment (currently divided into two domains: abulia/apatia including angedonia, asociality, and definitions of diminishing emotion intensity including stupefied affect and alogia [15, 16]), as well as disorganization including formal thinking disorders, inadequate affect, and disorganized behavior. Modern classifications, DSM-5 (American Psychiatric Association, 2003) and ICD-11, are based on the dimensional approach.

Symptoms of various dimensions may coexist in one and the same patient in different qualitative and/or quantitative combinations [15, 17–19]. Although discussions concerning the number of obligate dimensions are going on (two- [20], three- [21], five- [22], and multifactor [23] models of schizophrenia are meant), dimensional integration of symptoms seems to be most adequate for the assessment of multimodal data on possible biomarkers [24].

The work [25] may serve as an example of improving the quality of research conclusions and contribution to understanding etiopathogenesis of schizophrenia. The results of this work show alterations in fractional anisotropy in nine tracts of the brain white matter substantially correlating with separate domains of psychopathology. The same paradigm is also the basis for the RDoC project, in which the transition is implemented from the traditional nosological categories of schizophrenia to isolated phenomenological manifestations of symptom dimensions of this disorder [26] with clarification of stages of possible ways of psychopathology development: from genetic predisposition to physiological and behavioral manifestations [27].

## *Section 2*. Biomarkers of schizophrenia

### Concept of a biomarker

Delineating the concepts, it is necessary to make clear that a biological marker is a characteristic which is objectively measured and evaluated as an indicator of normal and pathological biological processes including responses to the therapeutic (pharmacological) intervention [28]. In other words, a biomarker may be employed as an indicator of the norm and pathology on the basis of changes of biological functions or serve as an indicator of alterations in the organism in the course of therapy^2^. Not only molecules (for example, receptors, nucleotides, or immunoglobulins) may be biomarkers, but electrophysiological indices or tomography findings as well. evidence agreement and experimental validation, analogous experience [30].

In medical practice, biomarker validity is usually confirmed by the evaluation of its sensitivity, specificity, positive and negative prognostic value [29]. Sensitivity and specificity of biomarkers is the ability to identify patient’s condition based on the marker analysis. Positive prognostic value means the probability that subjects with a positive test really have the disease, while negative prognostic value is the probability that subjects with a negative test do not really have the disease. A significant level of evidence is a threshold satisfying all Bradford Hill criteria proposed in 1965: strength of association, consistency, specificity, temporality, biological plausibility/gradient, coherence,

A diagnostic biomarker can identify only true positive cases rather than false negative, and this is the only way to determine the prevalence of some specific disease [31]. Besides, it must have sensitivity and specificity not less than 80% and positive prognostic value not less than 90%, moreover, it must be reliable, reproducible, non-invasive, inexpensive, and reproduced by at least two independent investigations [31].

### General directions of search for biomarkers

In spite of the fact that biopsychosocial paradigm of studying schizophrenia remains vital, the discussions about which factor plays a key role are gradually giving place to considering the mechanisms of their interaction at the current stage. At the same time, the dichotomy of studying schizophrenia pathogenesis is preserved in two aspects: from the standpoint of neurodegeneration (due to the effect of endogenous and exogenous factors) and from the standpoint of neuroontogenesis abnormalities (as a predisposition to the disease).

A neurodegenerative hypothesis implies slowly but steadily progressing destruction of neural tissue as the disease develops [32, 33], associated with the changes in the functions of neurotransmitter systems and also with neuroimmune impairment arising due to oxidative stress at the stages of psychoses or unfavorable effect of antipsychotic preparations [34, 35]. In order to confirm this hypothesis, longitudinal studies of the markers are required which may endure long-term measurable changes.

According to the neuroontogenetic hypothesis of schizophrenia [36-38], abnormalities leading to mental disorders are genetically inherent and/or are formed in the pre-, perinatal period, i.e. long before the debut or manifestation of psychotic disorders [39-41], in the form of motor, neurological, and behavioral deviations displayed at early age in children who later become ill with schizophrenia [42, 43]. Therefore, in order to test the neuroontogenetic hypothesis, it is reasonable to use markers being laid down at the early stages of the development, minimally changeable, and not subject to the impact of the external factors throughout later life. By the present time, the neuroontogenetic hypothesis of schizophrenia has been expanded to the theory of neuroontogenetic continuum demonstrating various domains of impairment development in the ratio with severity of psychic syndromes and the degree of cognitive disturbances associated with them (from very adverse in congenital mental incapacity up to relatively mild in bipolar affective disorder) [44].

Several international consortiums, whose researches meet the highest level of evidence: prospective analysis of cohorts which include thousands of patients examined in several independent scientific centers, are involved in overcoming possible disagreements and search for common ways of neuroontogenesis and neurodegeneration in schizophrenia [45, 46].

Methods of positron emission tomography (PET), single-photon emission computed tomography (SPECT), and magnetic resonance spectroscopy (MRS) may be used to study alterations in the cells for detection of current state biomarkers, while diffusion tensor imaging (DTI), structural and functional magnetic resonance imaging (sMRI and fMRI, respectively) may examine anatomical and functional changes, respectively [47, 48] and are suitable for checking both hypotheses in the process of cross-sectional and prospective studies.

Present methods of analysis of large-volume heterogeneous datasets and machine learning allows not only effective work within one modality but consolidation of the data from various modalities (for example, reflecting the current state of the cells and anatomical changes of the large brain areas) to seek within them complex interconnections which may be potential biomarkers. This approach will make it possible to retreat in future from the existing dichotomy of schizophrenia pathogenesis and consider neuroontogenetic and neurodegenerative processes simultaneously.

### Neuroimaging biomarkers of schizophrenia

Current studies of schizophrenia demand the development of criteria for objective complex assessment of structural and functional changes of the brain as a substrate of disease development. Lately, several complex investigations on the schizophrenic-type disorders (for example, [49, 50]) in order to find specific features of the disease and define the key zones involved in pathogenesis using MRI data, morphometry, tractography; brain activity by resting-state MRI findings and under cognitive load; metabolism by the data of PET, or concentration of neuromediators in gamma-aminobutyric acid (GABA) and glutamate obtained from the MRS data, and so on.

Neuroimaging examinations of patients with the diagnosis of schizophrenia with auditory hallucinations allowed one to achieve some understanding of the association of these psychopathological symptoms with the disturbances in specific brain areas and neural networks. Therefore, despite the diversity of symptoms and schizophrenia manifestations, in this section we will focus our attention on the topical studies of hallucinatory-paranoid syndrome of schizophrenia and highlight the mechanisms of its development. These studies have shown that auditory hallucinations are connected with functional and anatomical disturbances in the structures responsible for the areas of auditory perception, i.e. primary and secondary auditory cortex, and also in the structures responsible for speech production, i.e. opercular part and anterior insula of the inferior frontal gyrus. In addition to the alterations in the auditory and speech zones, changes in a number of other cortical and subcortical regions are also being discussed.

### Biomarkers of voxel-based brain morphometry

Neuroimaging studies of the brain structures using voxel-based morphometry have shown that auditory hallucinations are associated with the reduction of the grey matter volume in the superior temporal gyrus [51] which sometimes includes primary auditory cortex of the left hemisphere, middle temporal gyrus, and, to the less extent, regions not belonging to the temporal lobe [52]. The reduction of the volume in the temporal lobe is confirmed in the meta-analysis of nine similar investigations in which disturbances in the grey matter were studied in schizophrenic patients with auditory hallucinations [53]. In the work [54], the intensity of auditory hallucinations has been shown to be connected with the reduction of the grey matter of the superior temporal gyrus of both hemispheres including primary auditory cortex. The left superior-temporal region is known to process information connected with speech perception, i.e. with the recognition of phonological and semantic speech characteristics. The right superior temporal gyrus has a subthreshold effect of auditory and verbal information perception and is also involved in the processing of this information, especially emotional and prosodic aspects of the speech stimuli.

Some authors find intensive disturbances related to auditory hallucinations in the nonsensory parts including insula, anterior cingulate, posterior cingulate, and internal frontal gyri, thalamus, cerebellum, and precuneus [52, 53]. In their study conducted on a large sample of patients (n=99) with auditory hallucinations, Nenadic et al. [55] have shown relations between the intensity of auditory hallucinations and grey matter reduction in the left postcentral and posterior cingulate gyri, i.e. in the regions responsible for the integration of personally significant stimuli [56]. Volume reduction in the regions of the parahippocampal gyrus [57] and tonsil [58] verifies the idea that abnormalities in the limbic structures important for the processes of emotional regulation, i.e. beyond the speech zones, are closely connected with the occurrence of auditory hallucinations [51]. These data should be taken into account when building neurocognitive models directed to the explanation of auditory hallucinations. Despite some differences between the described works, revealing the disturbances in the auditory cortex and parts of the brain connected with speech gives the most reproducible results [52].

### Index of brain gyrification as a biomarker

In the meta-analysis [59], one can find a detailed overview of the current state of using gyrification index as a biomarker of schizophrenia. The analysis of insular cortex surface in patients with first-episode schizophrenia not receiving medication therapy made it possible to find the correlation between the gyrification of the cortex and presence of delusion and hallucinations [60]. The study [61] using the same methods but a larger patient sample (225 patients with schizophrenia spectrum disorders) has shown that the intensity of auditory hallucinations was connected with the dimensions of the insula surface zone. A more frequent similarity of gyrification in the appropriate Heschl’s gyrus (the area in the right hemisphere) was detected in patients with hallucinations compared with those without hallucinations and the healthy [62]. Thus, specific impairment of primary auditory cortex morphogenesis is detected in patients with hallucinations already at early stages of schizophrenia.

Advancement of technologies allows one to perform automated examination of cortical gyri over the entire cortex surface and to improve the reliability of measuring their complexity, variability, and three-dimensional contours [63]. This approach helped detect a marked decrease of the area of the speech cortex gyri in 30 patients resistant to medication therapy of auditory hallucinations in comparison with 28 healthy tested people [64]. Emergence of true or pseudohallucinations is related to the abnormalities in the morphology of the sulcus between the temporal and perietal regions of the right hemisphere manifested in the process of brain development [65].

### Tracts of the brain as a biomarker

The most important of the neuroimaging investigation techniques is diffusion tensor imaging (DTI) which provides the opportunity to identify special features of the brain white matter (WM) microstructure and the level of connectivity of various structure tracts in norm and its impairment in schizophrenia [66]. Still at the end of the XX and the beginning of the XXI century, microstructural anatomical abnormalities were found in the neural connections, synaptic contacts, and density of oligodendroglia in the prefrontal areas, callosum, and caudate nucleus of the postmortem specimens of the brain tissue [67]. These studies served as a basis for further works on the investigation of white matter pathology in schizophrenia.

The main disturbances in schizophrenia are observed in the neural network consisting of the frontal area, thalamus, striate body, and cerebellum [68]. Davis et al. [69], using the voxel-based method of measuring white matter volumes, have demonstrated that in schizophrenia, myelinization of axons responsible for the pathology of this substance is significantly impaired. In patients ill with schizophrenia, there is observed a reduction of fractional anisotropy in the corticothalamic tract whose fibers are connected with dorsomedial thalamic nuclei as well as the right superior temporal area, auditory integration zone, and the right auditory association area [66]. Additionally, disruption of the connections has been shown to be between the frontal, temporal, and parietal parts and the reduction of the intervoxel coherence (IC) index in patients with auditory hallucinations in the following structures: hippocampus, posterior parts and genu of the corpus callosum. The IC index in patients with the first episode of schizophrenia was reduced in the frontal, parietal, and occipital cortical areas [70] and also in the stem and corpus callosum, arcuate fasciculus, cerebellar internal capsule, and peduncles [71]. Federspiel et al. [66] confirmed the IC reduction in the enumerated structures and was also found in the left posterior cingulate gyrus which is actively involved in cognitive functioning. Studying schizophrenic patients with delusions, Oestreich et al. [72] have found in them a lower level of fractional anisotropy in the callosum, superior longitudinal fasciculus, arcuate fasciculus, and in the fasciculi of axons projecting from the cingulate gyrus to entorhinal cortex. Referring to the existing data on anatomical damages detected in schizophrenia and functional disturbances of the white matter in various brain structures (longitudinal and arcuate fasciculi, entorhinal cortex). Zhou et al. [73] consider the disrupture of connections between these structures and prefrontal cortex, which causes the main productive (delusion and hallucinations) and cognitive symptoms, to be most important.

In the work [74], it has been shown that patients with chronic and therapy-resistant hallucinations demonstrate reduced fractional anisotropy in the arcuate fasciculus due to the increase of productive symptoms and “magnetization transfer coefficient” values designating the increase of free water concentration due to the diminishing integrity of axons or glial cells. Growth of the “magnetization transfer coefficient” in the arcuate fasciculus is also observed in chronic patients with “voices inside the head”, which indicates the presence of specific connection of this coefficient with auditory hallucinations to the greater extent than with other productive or negative symptoms [75].

### Functional brain regions as a biomarker

There are several functional neuroimaging studies analyzing neuronal correlates of clinical characteristics of patients suffering from schizophrenia with auditory hallucinations. Vercammen et al. [76] investigated subjective physical characteristics (loudness and reality) of auditory hallucinations using metric assessment in the task defined as “reality discrimination task”, in which regions producing internal speech and perceptive parts were activated concurrently. The loudness of voices correlated with the activity reduction in the sulcus angularis of both hemispheres, anterior cingulate gyrus, left internal frontal gyrus and insula, and also in the left temporal cortex, while auditory hallucinations were associated with the reduced speech lateralization [76]. In the work of Raij et al. [77], subjective reality of voices correlated with the activation related to hallucinations in the left internal frontal gyrus and in its connection with the frontal gyrus and other cortical and subcortical parts including anterior cingulate cortex.

Two works [78, 79] studied emotional dysfunction in patients with auditory hallucinations. When emotional auditory stimuli were presented, patients with and without auditory hallucinations demonstrated increased excitation in the parahippocampal gyrus and tonsil compared to the healthy controls [78]. In a similar work, patients with auditory hallucinations demonstrated decreased activation of the tonsil and hippocampus when listening to emotional sounds in contrast to the patients without auditory hallucinations [79].

Activation of the secondary cortical zone during auditory hallucinations is also a frequently repeated result and was confirmed in the meta-analysis devoted to hallucinatory syndrome [52]. This work has shown that in hallucinations there is increased likelihood of activation in vast lateral frontotemporal networks including Broca’s area, anterior insular zone, precentral gyrus, frontal operculum, middle and superior temporal gyri, internal parietal lobule, hippocampus, and parahippocampal region. The studies, investigating cortex activation preceding the emergence of auditory hallucinations, have detected the reduction of activation in parahippocampal cortex prior to symptom occurrence, which is opposed to the excitement at the moment of hallucinations [80]. Other researches have shown that disturbances in neural networks of the frontal and temporoparietal speech zones play a great role in the emergence of auditory hallucinations [81]. Patients with auditory hallucinations demonstrate weakened activation in the temporal, cingulate, premotor, cerebellar, and subcortical parts involved in the internal speech and verbal imagination.

The research by Diederen et al. [82] have identified numerous zones linked to auditory hallucinations including internal frontal gyrus, insula, superior temporal gyrus, supramarginal gyri, postcentral gyrus, left precentral gyrus, internal parietal lobule, superior temporal area, and right cerebellum in patients with psychosis receiving no medication therapy. Applying similar experimental conditions to patients without medication treatment, Linden et al. [83] have discovered increased activation in the areas of speech recognition i.e. superior temporal gyrus, frontotemporal speech regions, and supplementary motor area, during auditory hallucinations and performance of the auditory imagination tasks as well. Interestingly, this activation in the prefrontal and sensory zones in auditory hallucinations differed radically from that in imagination due to the lack of voluntary control in hallucinations. During visualization, the activity of the supplemental motor zone preceded the excitation of the auditory zone, while at the moment of hallucinations both processes were going on at the same time [83]. Investigations, where participants were asked to remember whether they pronounced the target words, have shown reduced activation of the medial prefrontal cortex in patients with schizophrenia [84]. This region is considered to be involved in the assessment of stimuli which are of personal importance, and is selectively engaged in the solution of the tasks with these stimuli [55]. In the work [84], patients with and without auditory hallucinations were not compared directly, therefore impairment of the medial prefrontal cortex function may the basis of such a characteristic as dissociative disturbances in schizophrenia.

### Biomarkers of functional neural network connectivity

Functional connectivity between brain regions derived from the fMRI data is more and more often used to clarify disturbances in neural networks playing important role in the development of auditory hallucinations in schizophrenia. Functional connectivity reflects correlation between the dynamics of BOLD activity defined for two and more regions. In one of the first studies evaluating functional connectivity in the process of solving the tasks on sentence completion, the results showed its reduction between the left dorsolateral prefrontal cortex and temporal parts in patients with schizophrenia in comparison with the norm, and the correlation value had a feedback with the intensity of auditory hallucinations [85]. Mechelli et al. [86] investigated patients with and without auditory hallucinations using fMRI when they were performing the task on the assessment of the recorded speech (their own or someone else’s). Functional effect of one region on the other was assessed in compliance with stimulation conditions using the effective approach to connectivity calculation. In healthy and ill people without auditory hallucinations, the influence of the left superior temporal area on the activity of the anterior cingulate cortex was greater during perception of speech pronounced by others relative to their own speech. In patients with hallucinations, an opposite picture was observed. A later work [87] based on the task on speech identification (one’s own or someone else’s) demonstrates the same results, which, in authors’ opinion, proves the fact that functional connectivity between the medial prefrontal cortex (cortical regions along the central line responsible for the processes of self-observation) and the left superior temporal gyrus was impaired in schizophrenia in contrast to the norm. However, the difference between patients with and without hallucinations has not been assessed in this work.

There are works, in which the paradigm of implicit situation or resting-state has been employed. This approach provides the possibility of identifying spontaneous interaction of neural networks, resulting in auditory hallucinations as no specific task is being done at the time of data registration. Vercammen et al. [88] have calculated functional connectivity under implicit conditions for a selected “region of interest” located in the right and left temporooccipital zones, comparing patients at the time when they were experiencing auditory hallucinations with healthy participants. The group of patients with schizophrenia has demonstrated impaired functional connectivity of the left temporal-occipital zone with the right hemisphere analog of Broca’s area. Patients with more intensive auditory hallucinations were noted to have reduced connectivity between the left temporoparietal zone, bilateral anterior cingulate parts, and the tonsil.

Gavrilescu et al. [89] have explored interhemispheric functional connectivity between the primary auditory cortex and secondary auditory cortical zones at a resting-state in patients suffering schizophrenia with auditory hallucinations. A similar study was conducted with patients without hallucinations and healthy subjects. Functional connectivity was assessed using a resting-state fMRI in the selected “regions of interest”, determined for each participant by the functional activation maps in response to word listening. Patients with hallucinations have demonstrated reduction of interhemispheric connectivity relative to the two other groups.

Hoffman et al. [90] compared patients with schizophrenia spectrum disorders with auditory hallucinations with patients having the same diagnosis without auditory hallucinations and with healthy people. Functional connectivity was detected in the Wernicke’s area and the corresponding area in the right hemisphere. It formed a loop integrating the Wernicke’s area and the “region of interest” in the inferior frontal gyrus, while the putamen of the lentiform nucleus was significantly enlarged in patients with hallucinations against patients without hallucinations and the norm. Patients with and without hallucinations had a lower functional connectivity than in the control group in the Wernicke’s area in comparison with the corresponding region in the right hemisphere and also between the Wernicke’s area and anterior cingulate gyrus.

### Neurophysiological biomarkers of schizophrenia

The EEG technique has some advantage over MRI in greater accessibility and ease of use in clinics as well as in registration of “fast” changes of brain’s electrical activity. Components of evoked potential and functional connectivity, or coherence may serve as most specific electrophysiological indices for schizophrenia [91, 92]. Searching for and studying functions as well as interactions by the parameters of resting-state network connectivity, saliency, and executive control in patients with schizophrenia show similar features in the majority of works: perception impairment, selectivity of attention, selection of significant information in this pathology is linked to the reduction of interconnections of these networks [93].

In electrophysiology, perception disturbance like pseudohallucinations typical for clinical picture of schizophrenia finds its explanation in the theory of efference copy, according to which patients fail to assess stimuli to the inner and outer irritants. According to Ford [94], patients with schizophrenia do not have signs of efference copy during speech and internal speech characteristic of healthy people: there is no N1 wave of evoked potential, coherence between the motor and sensory speech centers is not available; this situation in its turn leads to the identification of the internal speech as external and is felt as “voices inside the head”.

Disturbance of the thinking process in the form of distortion of information selectivity or relevance is studied in detail on the fMRI findings. However, there are electrophysiological data on statistically significant differences in patterns of synchronization between patients from the target group and that of the control. Using the analysis of inter-frequency phase connectivity, the authors isolated functional frontotemporal networks with central and temporal components in theta and alpha frequency range which can reflect differences in relevance detection. Error in the assessment of stimulus significance in patients with schizophrenia is also confirmed in the theory of predictive coding.

Numerous works include analysis of differences at various stages of evoked activity in patients with schizophrenia and healthy subjects. Here we may distinguish researches directed to the study of disturbances of early components of evoked potential responsible for the analysis of stimulus sensory components and investigations devoted to the late stages of processing — semantic analysis, significance assessment. Despite the difficulties of isolating sensitive electrophysiological parameters using EEG and often contradictive research data, several basic features may be singled out which show persistent significant differences between normal groups and those with schizophrenia (see the Table). In clinical practice, there proved to be effective estimation of P300 component amplitude linked to the initial processing of stimulus significance, selective attention, and being essentially different in patients with schizophrenia and healthy controls.

**Table:** The main electrophysiological biomarkers obtained from EEG and their characteristics for patients with schizophrenia

**Type of biomarker according to EEG data**		Characteristic of biomarkers in schizophrenia relative to the healthy group
Components of evoked potential	Early components — P50, N100	Amplitude reduction, latency increase Peak inversion for emotionally significant stimuli
	Middle components — P300	Amplitude reduction, latency increase for auditory evoked potentials
	Late components — N400, P600	Latency elongation for complex stimuli
	Contingent negative variation (CNV)	Mismatch negativity (MMN) is smaller by amplitude for auditory evoked potentials
Functional connectivity	Coherence (evoked activity)	Reduction of coherence in patients with schizophrenia with predominance of negative symptomatology and its elevation in patients with predominance of positive symptomatology in case of low interhemispheric connectivity, and greater (relative to the norm) connectivity of the frontal and parietal regions, occipital areas between the hemispheres, temporal and parietal areas
	Inter-frequency connectivity	Connectivity reduction of theta and beta ranges, alpha and beta ranges
Dipole sources of evoked potential	For the component of evoked potential	Reduction of P300 amplitudes of temporal-basal dipoles corresponding mainly to P3b dipole. Intensive productive symptomatology correlates positively with the activity of the temporal-basal P300 dipole, whereas negative symptoms correlate positively with the activity of superior temporal dipole
	For MMN potential	In schizophrenia, switching from involuntary processes of auditory perception to voluntary changes, which is reflected in the bias of temporal dipolar sources of MMN component in the debut of the disease on the sources in the left cingulate gyrus
	For EEG rhythms	Patients with schizophrenia have significantly more dipolar localizations of alpha rhythm sources in clusters localized in limbic cortex and hippocampus. For theta rhythm, increase of dipole moment of the source is noted in clusters located in the regions of temporal, frontal cortex and hippocampus

Attempts were made to identify markers of schizophrenia by EEG based on machine learning. Thus, during informative features for machine learning selection, a phase-locking value method proved to be rather effective. The analysis, built on this method for various groups of mental diseases, has shown the main markers for schizophrenia in the frequency range of the alpha rhythm [95]. According to Kim et al. [96], machine learning can help define the main neurophysiological characteristics of patient with schizophrenia with a high and low score obtained by cognitive tests and with a high score in positive/negative symptomatology according to PANSS. The data obtained by these authors showed the link of the limbic system structures, the insular cortex in particular, and also features of differences in the theta, alpha-1, alpha-2, and beta-2 EEG rhythms in patients with different level of cognitive function preservation. Seven regions (connectivity centers) of phase-locking activity have been distinguished. The most precise classification of healthy people and patients with schizophrenia was shown by the values of frontal and parietal lobe connectivity, and then followed the connectivity of occipital regions, limbic system, temporal and parietal lobes in the descending order of significance.

Significant differences of connectivity values in the limbic system are also confirmed by other investigations without using machine learning [97]. When classifying patients with a high cognitive level and those with a low score, this index gave the highest accuracy inside the target group. The feature, which classified most precisely patients with positive and negative symptomatology predominance, is an indicator of connectivity of the frontal, temporal-occipital and parietal lobes, and insula. Upon the whole, functional connectivity in patients with the predominance of negative symptomatology and decline of cognitive functions is lower than in patients with predominance of positive symptomatology and safe cognitive functions.

As for the features isolated by the main EEG rhythms, power characteristics of theta and beta ranges were isolated most often from 11 frequency ranges when classifying the target and control groups with the help of machine learning algorithms. When intragroup classification of patients with schizophrenia according to the preservation of cognitive area was performed, the algorithm isolated the power of alpha rhythm as well as delta and (to the less degree) theta, beta, and gamma rhythms. Classifying by the intensity of predominance ofpositive or negative symptomatology, characteristics of alpha-rhythm band were selected most often, thereafter followed characteristics of delta, theta, beta, and gamma band rhythms in the descending order of significance. Connectivity of theta and beta ranges is described in detail for the healthy group in the process of solving cognitive tasks and perceiving emotionally significant information [98].

Researches, aimed at differentiating schizophrenia from depression by means of biomarkers derived from EEG, are interesting from the methodological point of view rather than practical [99], as the task of differentiating the diagnosis of schizophrenia and schizoaffective disorder from depression with a substantially different clinical picture, is usually more vital in the clinic. However, such investigations are also conducted. For example, the authors of the same work [99] obtained the markers discriminating schizophrenia from major depressive disorder by the parameters of cognitive evoked potentials, namely dipole sources, isolated by the LORET algorithm and related to both the intensity of positive symptomatology and the level of cognitive functioning. 28 features were selected for the sources, whereas 12 were selected for peak latency of evoked potentials N100 and P300a, and only 8 features — for the power amplitudes according to the rhythm ranges. The authors have concluded that the analysis of dipole sources is more informative for schizophrenia identification than other methods. Vázquez et al. [100] distinguished the markers according to two parameters of functional connectivity. Such characteristic as “generalized partial directed coherence” (GPDC) in the alpha rhythm range for the connectivity of channels O1 and O2 and in the theta rhythm range for the same channels was used for general connectivity of the EEG channels. For interchannel connectivity, “direct directed transfer function” (dDTF), the connectivity of channels T3 and C3 in the gamma rhythm range, O1 and C3, T5 and O2 in the range of beta rhythm, and Fz and O2 in the range of theta rhythm was considered to be the feature.

The approach based on the preliminary classification of schizophrenia by the profile of disturbances in the basic psychic functions, and then, using machine learning algorithms, search for objective markers of the state, including EEG, seems to be rather promising. Search for biomarkers using multimodal data, i.e. by microbiota, immunological blood indices, and EEG, has been described in the article [101]. In this study, some combinations — biomarker profile — have been selected for the group of patients with schizophrenia as a result of processing a large number of the complex parameters. Using machine learning, 34 parameters were selected as features. Several basic combinations were obtained from them. The most significant of them are listed below:

connectivity with the center in right parietal region (P4) in the range of alpha-2 rhythm (from 10 to 13 Hz) and the amount of monocytes;

clustering coefficient in the right temporal region (T6) in the range of EEG theta rhythms (from 4 to 8 Hz) and concentration of *Ruminococcus* in microbiota;

degree centrality in the range of beta-2 rhythms (from 20 to 30 Hz) in the right frontal area (Fp2) and white blood cell count;

degree centrality in the range of beta-2 rhythms (from 20 to 30 Hz) in the right occipital area (O2) and neutrophil to lymphocyte ratio.

Tikka et al. [102] used high-resolution EEG (256 channels) isolating from it other features: maximum and minimum amplitude of spectrum power peaks in delta (0−4 Hz), theta (4–8 Hz), alpha (8–12 Hz), beta (16–32 Hz), and gamma (32–64 Hz) ranges, and performing wavelet analysis in these rhythm ranges. The following regions of interest were selected: projection on the cortex of the left inferior frontal gyrus, dorsolateral prefrontal cortex (DLPFC) of the inferior parietal lobe (IPL), and superior temporal gyrus (STG). Using SVM method, the following features showing intergroup difference between patients with schizophrenia and healthy were distinguished: gamma-activity in the areas above the right IFG, left DLPFC, and right STG. The most accurate feature determining the subgroup with the predominance of positive symptomatology over the negative, according to the authors’ data, was the increase of delta rhythm power in the area of the left IFG.

Thus, we may conclude that EEG biomarkers may be distinguished by neurophysiological parameters such as functional connectivity characteristics, amplitude and latency of the evoked potential components, parameters of the rhythmic patterns, and dipole sources (see the Table).

### Immunological biomarkers of schizophrenia

Molecular genetic studies of schizophrenia have shown that an essential role in the development and progression of this disease is played by the disturbances of the systemic immune response and immune processes in the CNS, i.e. chronic neuroinflammation. It has been proved by examinations of the post-mortem brain, clinical, and genome-wide studies [103–107]. Association of immunological disorders with schizophrenia prognosis [108] and symptomatology character is being investigated. Thus, in exacerbation of the disease, increase in the blood levels of proinflammatory cytokines IL-8, TNF-α and acute-phase protein (C-reactive protein, CRP) is observed [105]. According to other authors, a high level of CRP is associated with a more severe course of psychosis in schizophrenia and subsequent decline of cognitive functions [105, 106].

Mechanisms of immune disorder impact on the pathogenesis of schizophrenia are being intensively explored (see, for example, the review by Malashenkova et al. [107]). The ways by which systemic immune disorders affect the immune processes in the CNS, stimulating neuroinflammation, have been described. They include active transport of cytokines across blood–brain barrier (BBB), activation of the vagus nerve endings in the inflammatory microenvironment, cytokines secretion by BBB endotheliocytes triggered by the inflammatory mediators in the periphery blood circulation, and others. Neuroinflammation contributes to the excessive activation of the complement component C4 in the CNS — one of the mediators of the reduction in the amount of synaptic connections in sick people. Besides, a disbalance of the immune processes in the CNS in neuroinflammation causes alteration of tryptophan metabolism in the direction of increasing synthesis of kynurenic acid (KYNA) acting as the main endogenic antagonist of glutamate NMDA receptors and promoting glutamatergic hypofunction in sick people. Interconnection between excessive activation of humoral immune response, serological signs of neurotropic infections, formation of autoantibodies to the brain proteins and schizophrenia are extensively being studied [104]. Suppositions on interrelation of neuroinflammation with structural brain alterations are presented in the literature [109]. At present time, efficacy of anti-inflammatory therapy in schizophrenia is investigated, however, the results of these researches are controversial [110].

In spite of the fact that, according to current concepts, a significant role in the development and progression of schizophrenia belongs to the disturbances of systemic immune response and immune processes in the brain, only a small number of works is devoted to the study of the links between immune parameters and clinical data in schizophrenia, and methods of machine learning in this field have not been systematically utilized [103–107]. According to the work [111], in which the analysis of the results obtained was conducted by the multiple regression method, the level of proinflammatory cytokine IL-6 has negative correlation with the cognitive function parameters in outpatients with schizophrenia (r=–0.395), whereas the levels of IL-2, IL-4, IL-10, IL-17A, TNF-α, IFN-γ are not associated with cognitive functioning.

The authors [112] have investigated interrelations of activation of the stress regulatory networks and immune response with cognitive disorders in schizophrenia using machine learning algorithms. The study included 37 patients with chronic schizophrenia and 35 healthy volunteers. Serum levels of the immune activation markers and markers of sympathoadrenal axis activator (TNF-α, IL-2, IL-6, IL-8, cortisol) were assessed by the PANSS scale. The complex of indices of cortisol, TNF-α, and IL-8 allowed identification of patients with chronic schizophrenia with the greatest sensitivity (over 90%) and specificity (over 90%). Besides, these values positively correlated with the intensity of cognitive symptoms according to the PANSS. The step-by-step linear regression analysis has shown that cognitive symptom intensity determined by PANSS correlated with the disease duration and indices of cortisol, TNF-α, and IL-8.

In the work [113], index reflecting disturbances of neuroimmune interactions was determined in 120 patients with schizophrenia and cognitive deficit and 54 healthy volunteers based on the blood level of immunological parameters (CCL-2, CCL-11, IL-1β, sIL-1RA, TNF-α, sTNFR1, sTNFR2) and the results of cognitive tests. Methods of partial least squares (PLS) and soft independent modelling by class analogy (SIMCA) were applied for statistical processing. Index obtained by PLS analysis explained 75% of psychotic symptom variability, aggressiveness, excitement, mannerism, and negative symptoms. The results of the SIMCA analysis, in the light of the authors’ interpretation, give reasons for distinguishing schizophrenia with neurocognitive deficit as a separate class of the disease.

A question of interconnection of immunological disturbances in schizophrenia with neurophysiological data is also insufficiently studied. In the world literature, only single researches on this topic are encountered. Thus, according to the paper [114], an increased level of IL-6 in outpatients with schizophrenia is associated with abnormal thickness of the grey matter in some regions of the cerebral cortex. The results were statistically processed by ANCOVA method (analysis of covariance). However, clinical interpretation of the results is difficult.

Of interest is the study [115], in which a model was created based on patients’ genotype predicting expression of complement components C4A and C4B participating in the pathogenesis of schizophrenia, and the result obtained was compared with the results of neuroimaging and cognitive tests. Negative associations were revealed between the predicted C4A expression and the results of some cognitive tests, as well as significant associations with the surface thickness and area of some cortical regions according to the MRI data.

The task of the work [116] was to find associations between the increase of the inflammatory marker level in blood plasma and inflammation subtype, cognitive disorders, and structural brain alterations in patients with psychoses. The study included patients with schizophrenia (n=50), schizoaffective disorder (n=29), bipolar I disorder with psychosis (n=61), and healthy volunteers (n=60). Blood serum level of inflammatory markers (IL-1α, IL-1β, IL-2, IL-4, IL-6, IL-8, IL-10, IL-12/ IL-23p40, IL-12p70, IFN-γ, TNF-α, TNF-β, CRP, Flt-1, VEGF, VEGF-C, VEGF-D, TGFβ-1, C4A), neuroimaging parameters (cortex thickness, volume of subcortical matter), clinical indices (the score by PANSS, YMRS, MADRS, BACS, spatial addition subscale of the Wechsler Memory Scale, dot pattern expectancy task, ER-40 test) have been studied. A combination of exploratory factor analysis and hierarchical clustering was used to identify inflammation patterns. The authors have shown that the levels of IL-6, TNF-α, VEGF, and CRP were statistically significantly elevated in psychoses. They also have established that the levels of separate markers and patterns of inflammation are associated with the results of neuroimaging, cognitive disturbances, symptom intensity. A group of patients with intensive systemic inflammation (7 markers with increased level in 36% of the examined patients in the main group and in 20% of the control group) was identified. These patients were noted to have statistically significantly worse results of cognitive tests on visual-spatial working memory and delayed response, as well as enlarged volume of hippocampus, tonsil, putamen thalamus, signs of increased cortical thickness as compared to the patients without the elevation of the inflammatory marker levels. The results of such works show good perspectives of more extensive interdisciplinary researches devoted to the study of interconnections between clinical immunological disorders and neuroimaging data in schizophrenia and the importance of developing new approaches to the interpretation of data obtained in these investigations.

Thus, the analysis of schizophrenia biomarkers allows us to conclude that in conjunction with a large data volume acquired from brain neuroimaging and immunological investigations and taking into account a complicated character of the clinical picture in schizophrenia described by numerous variables, application of machine learning methods in this field will give the opportunity to discover new scientific data on the mechanisms of the pathogenesis, disturbances of neuroimmune interactions of the disease, to gain an insight into the role of immunoinflammatory disorders and immunity disbalance in the multifactorial pathogenesis of schizophrenia. From a practical point of view, the results of data analysis performed by machine learning methods makes it possible to develop new panels of markers having diagnostic and prognostic value, design a prototype of the system supporting medical decision-making based on the selected features. To solve these tasks, it is important to establish interrelations between the factors influencing the pathogenic mechanisms (mediators and cells of the immune system), structural brain alterations (findings of neuroimaging), and clinical manifestations of the disease.

## *Section 3*. Utilizing data analysis and machine learning methods for the task of biomarker searching

### Specificity of data and their preprocessing

Nowadays, methods of machine learning and intelligence data analysis are utilized in medical researches for detecting diagnostic biomarkers and predicting the results of treatment using neuroimaging data, electrophysiological and immune data collected for target groups or healthy volunteers [117]. One of the problems of successful application of automated machine learning-based diagnosis in clinical environment is to provide the best possible quality of the original signal used for decision-making by the model. Data cleaning to remove the noise connected, for example, with scanning, is considered to be necessary, since the noise like this reduces sharply the effectiveness of recognition making impossible identification of neuroimaging markers of psychiatric disorders such as schizophrenia. In order to clean the data, it is necessary to use the reliable schemes of their preliminary processing which include a comprehensive testing of space-time constituents of the original signal and identification of the appropriate components of the desired signal as it is proposed in paper [118].

A small size and heterogeneity of samples of separate scientific groups is a serious problem. Currently, several projects of augmented open-source databases are being developed which contain not only verified clinical data and case histories but also neuroimaging data in a unified format and some modalities of data of “wet” biology (biochemical and immunological blood analysis, genotyping results) see, for example, http://schizconnect.org/.

### Feature extraction and classical models

Apart from the problem of data cleaning, there is one more important issue: analysis of multimodal brain data, which is connected with their large dimensionality. For example, when using MRI, standard dimensions of voxels are within 0.5−2.0 mm^3^ in case of structural imaging (which gives ~10^7^ voxels for the entire brain volume). MR image consisting of a large number of small-size voxels has a higher spatial resolution and therefore larger dimensionality. The same is true for EEG records, which usually consist of tens of time-series (one for each channel) and thousands of measured values for each electrode. In order to avoid “the curse of dimensionality”, methods of machine learning are usually applied to the features of smaller dimensions extracted from the raw data with the help of feature extraction procedure. These procedures are often included into the preprocessing stage.

Examples of such low-dimensional features may be structural and morphometric parameters (volume, thickness, curvature) of the anatomical areas selected from MR image, which together form a feature vector. For instance, the FreeSurfer program for processing MR images divides them into the areas corresponding to the selected anatomical atlas, computes 7 volumetric characteristics for each cortical area and 9 geometric characteristics of subcortical regions.

A promising way to informative feature extraction for clinical neurology and psychiatry is the calculation of functional brain connectivity, i.e. some mathematical representation of the functional brain architecture, which is determined by a set of vertices and edges. Patterns of abnormal functional connectivity may serve as an indicator of some dysfunction. Some models of functional connectivity include probabilistic graphical models [119] and sparse low-rank functional brain networks (FBN) [120]. When presenting brain regions in the form of vertices based on some brain atlas, it is possible to build graphical representation of interactions between these regions. Once the graph is built, the main graphical descriptions (features) are extracted from it (for instance, degree of a node and global network efficiency) for further employment in the patient classification tasks. Some tasks show the efficiency of the approach in case of using high-quality data; this method may also provide interpretable results from the physiological point of view [121].

### Models of deep learning

The abovementioned approach with the extraction of informative features possesses some disadvantages: apart from additional resources for data preprocessing, the researcher is required to understand what features are to be extracted. If some attributes were not extracted from the initial data, they will not be used in any way in building the models of machine learning, which may have a negative effect on the precision and robustness of these models. Deep learning (DL) is an alternative to this approach where high-dimensional initial data may be fed to the model input without any preprocessing: full-size MR image, entire EEG record, or a complete set of immunological data.

Convolutional neuronal networks have shown good results of classification in the tasks related to a number of psychoneurological diseases [122]. Two different architecture of a three-dimensional convolutional network were proposed in this study for solving the problem of differentiation of patients with Alzheimer’s disease from healthy control on the basis of their structural MR images. The deep learning models proposed by the authors have demonstrated similar accuracy: 0.88 and 0.87 ROC AUC, respectively, in the task of classifying patients with Alzheimer’s disease relative to those with a mild cognitive impairment and a healthy group. These results are comparable with earlier approaches using preliminarily extracted low-dimensional features. The result shows that convolutional networks may be applied directly to the raw neuroimaging data without much loss of classification performance, which makes it possible to omit the stage of preprocessing.

As applied to medical tasks, recurrent neural networks may be effectively employed for the analysis of temporal rows such as EEG data or fMRI. Dvornek et al. [123] used recurrent networks of LSTM type to search for biomarkers of autistic spectrum disorders by the resting-state fMRI data. The authors have demonstrated classification accuracy of about 0.69 in the task of classifying patients with this disorder and healthy people. Moreover, the authors made a comparison with earlier approaches on a large set of data available for autistic spectrum disorders, which showed that recurrent neural networks surpassed in accuracy the existing results by 9%. However, the given recurrent network receives as input data temporal rows obtained from the regions of interest which must be pre-extracted from the raw fMRI data, which may be also considered, to some extent, feature extraction albeit highly dimensional.

Dakka et al. [124] have proposed the model combining the merits of recurrent and convolutional networks to classify patients with schizophrenia by initial fMRI data in the form of a sequence of three-dimensional MR images. The authors considered several configurations consisting of several convolutional blocks with 3D filters and two layers of LSTM network. The most successful of these models showed classification accuracy of 0.65 and significant improvement of performance as compared to the basic prediction methods such as support vector machines (SVM) and others.

### Multimodal data integration

At present, numerous works are devoted to multimodal and multitask investigations in machine learning. Aggregation of multimodal data may provide machine learning models with a large volume of information about every subject from the sample as compared to unimodal data and will also allow the selection of cross-domain information and complex relations between various types of data unavailable in unimodal studies.

Different architectures are used for integration of multimodal data: deep confidence networks, convolutional, and recurrent neural networks. Recently, the so-called transformers became widely used: Transformers, Multimodal transformer (MMT) [125], LayoutLMv2 [126], DALL-E [127], Perceiver [128], Perceiver IO [129], OmniNet [130]. Deep neural networks of this kind enable identification and encoding of comprehensive interconnections between data and reduction of their dimensionality.

An example of the architecture, where multimodal data are integrated by means of deep confidence networks, is considered in the paper by Suk et al. [131] for solving the task of diagnosing Alzheimer’s disease. A neural network receives data from two modalities, MRI and PET, and thereafter its vector representation is built for each modality. Further, these representations are integrated and fed to the input of the next linear layer for building multimodal representation. The article [132] describes diagnosing of Alzheimer’s disease at its early stages using multimodal data (MRI, genetic, and clinical data). At the first stage of processing, intermediate compressed representations for each type of data are selected: 3D convolutional neural networks are used for MRI, autoencoders — for genetic and clinical data. Further, the intermediate features are transmitted to the linear layer for classification by the stages of Alzheimer’s disease.

The authors [133] believe that application of multimodal data in mental diseases may provide the research with more information on patients’ individual characteristics and give a clue to the understanding of missing links in psychiatric disorders. The article discusses an example of synergy from simultaneous analysis of data from different modalities. The authors consider features from several modalities: e.g. hippocampus volume and default mode network (DMN) using fMRI data. In this case, to calculate correlation of one modality (hippocampus volume) with the activity of all brain voxels in another modality and then to test the differences between the groups relative to these correlations is not the same that to separately estimate which volumes of the brain show group activity alterations and which regions in the DMN network demonstrate group differences. The first variant may be considered as data integration since both datasets are used for the assessment of the joint result. This approach improves in many cases the capability of differentiating patients and the control group.

In the articles [134, 135], it is reported that multimodal MRI data allow one to make some original conclusions on the key clinical aspects of schizophrenia. Many psychopathological studies have already indicated that there is an interconnection between the structure and function of the brain in mental disorders. The authors [136], in particular, discovered three regions including thalamus, anterior cingulate, and inferior parietal gyri which showed how structural and functional disturbances are connected with attention in schizophrenia.

In the articles [137, 138], the authors describe integration of multimodal data to classify healthy subjects and patients with schizophrenia. In the first paper, for solving this task, functional and structural MRI are aggregated by the deep models and mechanism of attention which make it possible to identify complex interconnections between the features of various modalities. In the second work, a multimodal deep learning models which integrate genetic data (single nucleotide polymorphism), structural, and functional MRI are used. Data integration improves the model quality from the accuracy of 81% (which is inherent to the best unimodal fMRI model) to 88%, and that shows the efficiency of using this approach.

Apart from solving multimodal tasks, creation of the architectures capable of performing the tasks both on separate data modalities and multimodal data are recently being actively developed. OmniNet, Perceiver, Perceiver IO may be referred to these architectures. Perceiver and Perceiver IO may build representations for arbitrary information and arbitrary tasks. Perceiver is a transformer-based architecture using attention principles which may input any data such as images, video, audio, point clouds, and their multimodal combinations without any primary transformations. It uses the attention mechanism to collate input data from different types of modalities with a fixed-size latent space which is further processed by a deep network with full attention. This process makes the main part of the network processing independent of the dimension and modality of the input data, which permits its scaling up to the large and multimodal data.

### Interpretability of machine learning models

In medical diagnosis, interpreting the prognosis for deep neural network models is an especially important and necessary part of learning and validation processes. It is explained by the requirement of model transparency for its practical use in health care: decisions taken by it must be clear to clinicians of various specialties. In particular, when interpreting medical images, there arises a difficulty between how well the method can create explanations and how well the explanation corresponds to the image areas being interpreted. Existing techniques are often capable of managing only one task.

Let us consider an example of interpreting model predictions on neuroimaging data. Multiple pertubations method is considered to be one of the classical methods of interpretation of model predictions [139]. This method is based on the detection of changes in the output data (predictions) in controlled perturbations in the output samples and creates, as a rule, blurred maps with low resolution. Explanations of the model construction based on the gradient (CAM [140], Grad-CAM [141], Guided Backpropagation, Deep Taylor [142], DeepLIFT [143], LRP [144]) ignore the features with a relatively low discriminative power and select only those possessing high significance, or vice versa, are restricted by assigning an estimator without large-scale spatial coherence to each separate pixel of the image. More state-of-the-art modifications, LIME [145], RISE [146], XRAI [147] employing “superpixels” (group of pixels, cognitive fragments) as a base for explanation, add flexibility, but are too expensive in terms of computation.

In recent time, approaches to simultaneous classification and feature attribution are being developed using a shared latent space of attributes with a classification layer: VA-GAN [148], ICAM [149]. These methods imply learning on the basis of generative networks being not always successful on a small data sample of medical tasks. Besides, generation of significance maps of brain pathology is not enough for clinical application, since the dynamics of the disease is very important for diagnosing (for example, which areas are affected by atrophy, how rapid it is developing, and so on) which is impossible to define from one temporal section, i.e. by static data.

Popularity of classical methods such as Grad-CAM or meaningful pertubations suggests that decisions based on the regions (distinguishing the object area) allow one to achieve a higher level of interpretability, i.e. give explanations which are easier for a man to understand. In case of neuroimaging data, it may be, for instance, a set of anatomical brain regions. However, it leads to the loss of spatial precision of explanation, which is reflected on the medical images, e.g. human brain or lungs, by the selection of too large zones, i.e. by low specificity and informativity for clinicians. Therefore, searching for a method capable of generating simultaneously precise and interpretable explanations and suitable for data specificity remains a burning problem to be solved for such tasks as diagnosis of schizophrenia.

## Conclusion

Despite a wide spread and social importance of schizophrenia, decades of researches, and numerous publications of separate scientific groups, diagnosis and prognosis of the disease have been based mainly on observation and questioning, interpretation of which may be subjective. Several factors may explain this situation. From the point of view of clinical aspects, solution of fundamental and practical tasks in diagnosing schizophrenia is complicated by ambiguity in the agreement of diagnostic criteria of schizophrenia on the one hand: the state of the same patient may correspond to the criteria of several disorders or not correspond to the criteria of a definite disorder in terms of the predominant categorical approach, and on the other hand, by phenomenological similarity of schizophrenia symptoms: it is often difficult to differentiate similar negative symptoms from cognitive disorders in this disease. Moreover, the similarity of symptoms manifestation is complicated by the fact that each of them may vary in severity and intensity. Presence of such dilemmas even leads to the proposals to abolish the term “schizophrenia” excluding it from the list of diseases. Some authors believe that only transition to the dimensional approach when, on the basis of factor analysis of the main manifestations of schizophrenia spectrum disorders, dimensions (domains) of their phenomenology or symptomatology are distinguished, may significantly improve the quality of solving both fundamental and practical tasks of psychiatry.

Each year, the number of works devoted to the search for biomarkers of schizophrenia by different modalities of laboratory investigations is growing. The described heterogeneity of the disease and frequent symptom similarity result in detection of a large number of weakly informative biomarkers with low clinical significance. For example, according to the neuroimaging data, even in the narrower selected paranoid-hallucinatory syndrome, multiple structural and functional disturbances in the cortical regions and subcortical structures have been found. Sometimes the results of the research groups contradict each other and are not reproduced. As mentioned above, this may be connected with the heterogeneity of the clinical picture, impact of some factors such as the age of the disease onset, course dynamics, medication therapy, concurrent development of negative symptoms. Besides, many investigations have shown that structural changes, for example, the volume of grey matter in the cerebellum, subcortical structures, cortical structures, as well as alterations in the functional connectivity of the resting-state neural networks may be biomarkers of schizophrenia. According to some findings, descriptions of the structural connections between the brain regions, or connectomes (structural connections acquired from the tractography (DTI) data and functional obtained from fMRI) appeared to be even more informative for the diagnosis than local characteristics of these regions. The described MRI biomarkers and specificity of changes in the EEG parameters may be input parameters for classification tasks in machine learning methods.

Despite existing evidence of availability of neuro-inflammation and immune disorders in schizophrenia, their spectrum, causes, association with neuro-physiological alterations are insufficiently studied. Application of the data on the connection of immunity changes with the character of symptomatology and their impact on the prognosis in clinical practice remains rather limited for the present, and as a rule, criteria for interpreting the results of investigations with a large number of the examined parameters are not available to clinical specialists, which promotes publication of the papers devoted to the analysis of the results only for separate immunity indices not sufficiently reflecting the state of the immune system. It should be also noted that in general there are few interdisciplinary studies of schizophrenia with the analysis of data of clinical investigations and neuroimaging together with the results of immunological studies.

The state of immunity and inflammatory indices in schizophrenia are being intensively studied including the analysis of large datasets of interdisciplinary researches in order to identify prognostic markers for various variants of this disease. At the same time, the application of the results of these researches in clinical practice is a problem due to the lack of algorithms giving a clear interpretation of these results to clinicians. However, such algorithms are necessary taking into consideration differences in the approaches to the analysis and representation of the results in biomedical sciences and neurosciences and sometimes an abstract character of the results of mathematical processing of large data sets from the clinical standpoint. Their creation is significant for fundamental science since it will allow the achievement of a deeper understanding of the role of immunoinflammatory disorders in the multifactorial pathogenesis of schizophrenia and is also vital from the practical point of view for further development of criteria of the prognosis assessment, new personalized approaches to prevention and therapy based on clinical, neurophysiological, and immunological examination.

Finally, from the viewpoint of the data science, noisiness, heterogeneity, and large dimensionality of multimodal laboratory data and small sample sizes in the investigations of separate scientific groups do not always permit effective application of conventional methods of data analysis and machine learning in the diagnostic tasks and search for stable biomarkers. Presently, international consortiums create open supplemented databases of patients with various data modalities, although to repeat successes achieved by machine learning (especially deep learning) in other fields, it is necessary to evolve both methods and models built on the highly dimensional unimodal data and new ways of aggregating data of different modalities.

Implementation of the obtained models and methods into real clinical practice is very difficult without their interpretability for clinicians; therefore, a separate direction of researches in the field of artificial intelligence and machine learning may be the development of methods for interpreting sophisticated models built on multimodal data. Moreover, application of machine learning data synthesis techniques reflecting current state of the cells and anatomic alterations of the large brain regions will allow one to step away from the existing dichotomy of schizophrenia pathogenesis and to consider neuroontogenetic and neurodegenerative processes simultaneously which will be certainly a great step forward for psychiatry as a science.

## Footnotes

1 Clear illustration of the contradictions between classifications is a controversial position of catatonia which is presented in the ICD-10 as a category of catatonic schizophrenia (F20.2) although the genesis of this syndrome includes specific pathological processes not intrinsic to schizophrenia. These remarks are also true for a wide spectrum of affective disorders which are found in section F20.4 — post-schizophrenic depression and F25 — schizoaffective disorder in the ICD-10.

2 For example, blood glucose level is a key point for diabetes diagnosis as its indices are used to establish diagnosis, make prognosis, develop the scheme of therapy.
